# Recurrent Inflammatory Myofibroblastic Tumor of Larynx Harboring a Novel THBS1::ALK Fusion

**DOI:** 10.1155/2024/4937501

**Published:** 2024-08-14

**Authors:** Namra Ajmal, Stacey M. Gargano, Ujwala Gosavi, Madalina Tuluc

**Affiliations:** Department of Pathology and Genomic Medicine Thomas Jefferson University Hospital, Philadelphia, PA 19107, USA

**Keywords:** ALK fusion, IMT, Inflammatory myofibroblastic tumor, larynx, THBS1

## Abstract

Inflammatory myofibroblastic tumor (IMT) is a rare soft tissue tumor primarily occurring in the abdominopelvic region of young patients, and it is characterized by spindle-shaped myofibroblasts, or fibroblasts surrounded by inflammatory infiltrate. Herein, we report a case of a 24-year-old male with a firm submucosal mass in the anterior right vocal fold diagnosed as an IMT that recurred 14 months later. The tumor demonstrated a novel THBS1::ALK fusion containing Exons 1–7 of the thrombospondin 1 (*THBS1*) gene fused to Exon 19 of the anaplastic lymphoma kinase (*ALK*) gene via next-generation sequencing with the NextSeq sequencer. The fusion of *THBS1* to *ALK* potentially results in increased expression and constitutive activation of the ALK kinase domain. These findings not only broaden the repertoire of known *ALK* fusion partners implicated in tumorigenesis but also provide a novel avenue for investigating the etiology of recurrent IMT by considering this fusion event as a causal factor. To our knowledge, this is the second case of IMT of the larynx with this novel mutation reported in the literature and the first such case with a detailed description of this specific fusion and clinical recurrence.

## 1. Introduction

Inflammatory myofibroblastic tumor (IMT) is a spindle cell neoplasm mostly affecting children and young adolescents. According to the 2020 “WHO Classification of Soft Tissue Tumors,” it is included within the category of “fibroblastic/myofibroblastic tumors” of the “intermediate (rarely metastasizing) type” [1, 2]. As the name describes, it contains myofibroblasts/fibroblasts in a mixed-rich inflammatory background [3]. While the prototypical organ affected is the lung [4, 5], IMT can occur in any extrapulmonary site where myofibroblasts are present, and the repair process can happen. These sites include the genitourinary tract (such as the bladder, prostate, urethra, and kidney), the gastrointestinal tract, the abdominal cavity and mesentery, skin, lymph nodes, breast, endocrine organs, head and neck, and the central nervous system [4]. Previously, IMT has been known by various terminologies, such as inflammatory pseudotumor, plasma cell granuloma, and inflammatory myofibroblastic sarcoma. These names primarily described the lesion's features and reflected the uncertainty about its true biological nature [4, 6]. However, advances in molecular analysis have aided in identifying IMT as a clonal process primarily characterized by the activation of receptor tyrosine kinases (RTKs). Studies have reported that 50%–70% of cases have alterations in the anaplastic lymphoma kinase (*ALK*) gene located on Chromosome 2p23 [7]. For this, ALK immunohistochemistry (IHC) is the most helpful marker as the neoplastic spindle cells' reactivity with ALK IHC corresponds strongly with the presence of a clonal *ALK* rearrangement.

Herein, we describe the clinical, histological, and molecular features of a 24-year-old male with recurrent IMTs of the larynx. The patient presented with hoarseness and dysphonia and underwent two surgical resections, followed by Kenalog 40 injections. The histological examination of both specimens showed a low-grade spindle cell lesion consistent with IMT. Interestingly, a novel thrombospondin 1 (*THBS1*) fusion with *ALK* (THBS1::ALK fusion) was identified through next-generation sequencing, providing new molecular insight into the pathogenesis of this rare tumor.

## 2. Materials and Methods

### 2.1. IHC

Immunohistochemical stains using the following commercially available antibodies were performed using the Ventana Benchmark Ultra platform (Ventana Medical Systems Inc.). All the antibodies below were commercially prediluted. ALK1 (ALK01), AE1/AE3 (AE1/AE3/PCK26), S100 (4C4.9), SOX-1 (SP267), CD34 (QBEnd/10), calponin-1 (EP798Y), SMA (smooth muscle actin) (EP192), p63 (4A4), and neurofilament (2F11). All positive and negative controls showed appropriate staining.

### 2.2. Next-Generation Sequencing

Total nucleic acids (RNA) were extracted from formalin-fixed, paraffin-embedded (FFPE) lesional spindle cells using the QIAamp DNA FFPE Kit (Qiagen, Hilden, Germany). The detection of fusion genes was performed using the Archer® FusionPlex® Pan Solid Tumor Panel (Diagnostica Longwood, Zaragoza, Spain), which used reverse transcription of RNA from the specimen followed by anchored multiplex PCR to amplify sequences from fusion RNAs. Amplicons were sequenced on a NextSeq 550Dx next-generation sequencer (Illumina, San Diego, CA, United States). Sequencing data analysis was carried out using Archer Analysis 6.0 software (ArcherDX, Boulder, CO, United States).

The RNA fusion panel is designed to detect all gene fusions involving 99 oncogenic genes. For the *ALK* gene, all gene fusions to Exons 2, 4, 6, 10, 16–23, and 26 [NM_004304] are detected. This assay is sufficiently sensitive to detect fusions present in at least 700 RNA copies in the specimen. The genomic coordinates reported with the results refer to the human reference genome version hg19.

## 3. Case Presentation

**Figure 1:**
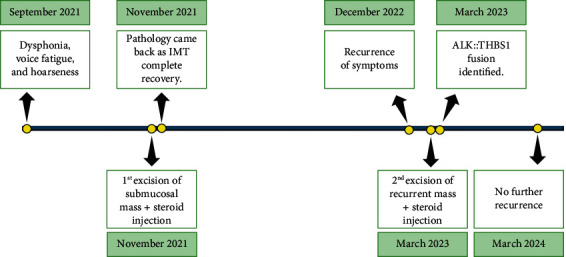
Timeline of events from initial presentation to present.

A 24-year-old man presented with dysphonia and hoarseness of voice, persisting for 6 months, accompanied by voice fatigue after prolonged use. He denied reflux symptoms, dysphagia, dyspnea, fever, or chills. Laryngoscopy with stroboscopy and dynamic voice evaluation revealed a submucosal mass involving the anterior portion of the right vocal fold and incomplete glottic closure. The initial clinical impression was of a submucosal cyst. Direct microlaryngoscopic examination revealed a firm submucosal nodule extending deep into the muscle, which was completely excised in multiple fragments, and Kenalog 40 was injected. The patient recovered well after surgery. Thirteen months later, the patient experienced a recurrence of hoarseness of voice. Microdirect laryngoscopic examination revealed similar findings: “a firm submucosal nodule that extended deeply into the muscle.” MRI neck and soft tissue only showed nonspecific postsurgical changes. The recurrent nodule was then surgically excised in multiple fragments (RO), and subsequently, Kenalog 40 was administered. No chemo or radiotherapy was administered (Figure 1).

**Figure 2:**
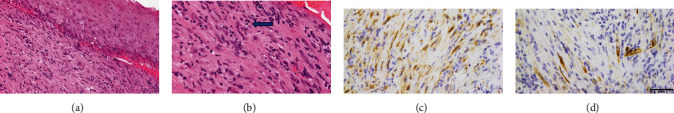
(a) Section showing benign squamous mucosa with rare inflammatory cells. Beneath squamous mucosa, fascicles of spindle cells are present (haematoxylin and eosin stain, magnification ×200). (b) Microscopic sections show a dense spindle cell proliferation associated with acute inflammatory (arrow) and lymphoplasmacytic infiltrate (haematoxylin and eosin stain ×400). (c) The spindle cells show diffuse cytoplasmic granular staining for anaplastic lymphoma kinase-1 (ALK1), (immunohistochemistry staining ×400). (d) The spindle cells show focal staining for alpha-smooth muscle actin (SMA) (immunohistochemistry staining ×400).

Initially, four pink–red soft tissue fragments ranging in size from 0.1 to 0.5 cm were received in the surgical pathology laboratory. Microscopically, the biopsies consisted of squamous mucosa with underlying submucosal proliferation of bland spindle cells arranged in fascicles in a background of myxoid stroma with mixed inflammatory infiltrates including neutrophils, eosinophils, lymphocytes, and plasma cells (Figure 2). No mitotic activity or necrosis was present. IHC showed that the spindle cells were diffusely and strongly positive for ALK, focally positive for capital SMA and calponin (Figure 2), and negative for cytokeratin AE1/AE3, p63, S-100, SO-10, STA-6, CD-34, and neurofilament. The characteristic morphology and immunophenotype support the diagnosis of IMT of the larynx.

After the second resection, multiple tan-pink to red tissue fragments measuring 0.7 × 0.3 × 0.1 *cm* in aggregate were received. These fragments showed histological and immunohistochemical similarities to the initial specimen and were confirmed as recurrent low-grade spindle cell lesions consistent with an IMT.

**Figure 3:**
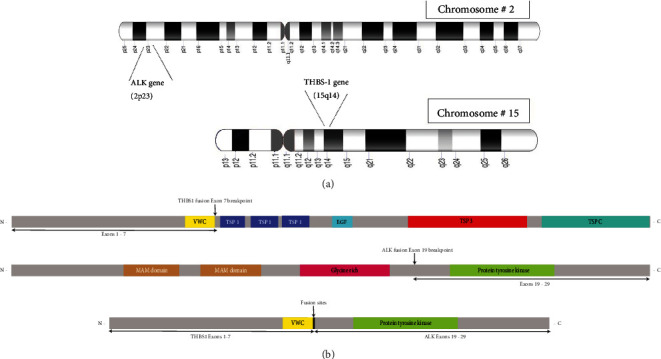
(a) G-banding ideograms of Chromosomes 2 and 15. The location of the THBS1 and ALK genes on respective chromosomes. Molecular characterization of FFPE sample from the larynx, right vocal fold mass. (b) NGS revealed an in-frame novel THBS1-ALK fusion with 5′ partner (Exons 1–7) of THBS1 and 3′ (Exons 19–29) of ALK. VWC, von Willebrand factor type C domain (318-372); TSP_1, thrombospondin Type 1 domain (383-428); EGF, calcium-binding EGF domain (588-625); TSP_3, thrombospondin Type 3 repeat (727-762); TSP_C, thrombospondin C-terminal region (972-1169); MAM-MAM domain, meprin/A5/mu (285-426); Gly_rich, glycine rich protein (733-960); Pkinase_Tyr, protein tyrosine kinase (1117-1382).

Molecular testing was subsequently performed, which identified a novel THBS1::ALK fusion (Figure 3). The fusions of Exons 1–7 of the *THBS1* gene and Exon 19 of the *ALK* gene were in-frame at the exon–exon boundary. The 5′ partner fusion sequence started from the end of Exon 7 of the *THB1* gene (chr 15; NM_003246.3; breakpoint 39,877,764), and the 3′ partner sequence was at the start of Exon 19 of the *ALK* gene (chr 2; NM_004304.4; breakpoint 29,448,431).

At the most recent follow-up, 12 months after surgery, the patient was doing well and had significantly improved his voice quality.

## 4. Discussion

In the head and neck region, IMT is a rare occurrence, and it is even more uncommon in the larynx. In addition to the larynx, other sites affected in the head and neck region are the oral cavity, pharynx, maxillary sinus, parotid gland, orbit, and bones (especially the mastoid) [7, 8]. In 1995, Coffin et al. reported 12 cases of head and neck IMTs, including three in the larynx, out of a total of 84 extrapulmonary cases [9]. In the same year, Wenig, Devaney, and Bisceglia presented a series of eight cases involving larynx [4]. Alhumaid et al. [10] reported 33 cases of laryngeal IMT, including the abovementioned cases. However, as expected, these studies were more focused on clinical and morphologic features [10]. Now, with more than 50 cases of laryngeal IMTs reported in the literature, a recently published paper by Kerr et al. in 2021 did a comprehensive study with IHC and molecular analysis of 13 head and neck IMTs, including seven laryngeal IMTs [7].

Laryngeal IMT occurs most commonly in the glottis, followed by the subglottis, and rarely in the supraglottis [4, 10, 11]. Presenting symptoms vary by location, with hoarseness (74%) being the most common. Stridor (29%), dyspnea (22.5%), globus sensation (16%), cough (6%), apnea (3.2%), and respiratory failure (3.2%) have also been reported [10]. Adult and pediatric populations are equally affected [3, 12–15]. Interestingly, two reported laryngeal IMTs had systemic manifestations—one patient had weight loss, fever, and raised ESR, while another had hypochromic microcytic anemia and thrombocytosis, both of which resolved after primary tumor excision [16, 17]. Smoking, trauma, viral/bacterial exposure, and immunologic host response have been proposed as possible causative agents [1, 16, 18].

Despite their benign behavior, IMTs are known to exhibit local aggressiveness and have a high propensity for recurrence. Recurrence rates vary depending on the anatomic site, ranging from less than 2% for lung-confined tumors to 25% for extrapulmonary lesions such as intra-abdominal organs or the head and neck. Distant metastasis is exceptionally uncommon [9, 12, 16]. Head and neck IMTs have a recurrence rate between 10% and 20% after surgical resection [19]. In the larynx, recurrence rates are reported between 8% and 18% (Table 1). In the realm of academic literature, it is widely acknowledged by authors that laryngeal recurrences primarily arise from incomplete excision of the tumor owing to its challenging surgical location [11, 20]. Also, no details regarding surgical margins were exhaustively documented in the recurring cases [21]. Advancements in the understanding of the indolent nature of the tumor have allowed for more conservative treatment approaches, such as reexcisions, radiotherapy, and recently, the use of ALK inhibitors, instead of the total laryngectomy that was used as a treatment option 25 years ago [4]. Also, real-time frozen sections can aid in achieving negative resection margins [14, 22].

The *ALK* gene located on chromosome 2p23.2 encodes a RTK that is only expressed in neural tissue under normal conditions [21] (Figure 3). *ALK* rearrangement has been identified in more than 50% of IMTs [28] and around 75% (21/27) of laryngeal IMTs [7, 29]. Besides IMT, the *ALK* gene is subject to genomic rearrangements across a range of malignancies, including anaplastic large cell lymphoma (ALCL), diffuse large B cell lymphoma, glioma, non–small cell lung cancer (NSCLC), colorectal, breast, ovarian, and esophageal cancer. Over 30 fusion partners have been identified, including *TPM3*, *TPM4*, *CLTC*, *RANBP2*, *ATIC*, *CARS*, *SEC31L1*, and *THBS1* [28, 29]. Most *ALK* gene partners provide a strong promoter and an oligomerization domain, resulting in the oncogenic activation of ALK kinase [30]. This leads to the production of a persistently activated chimeric fusion protein that further activates several downstream signaling pathways, including the RAS/MAPK pathway, the JAK/STAT pathway, the PI3K/Akt pathway, and the phospholipase C-*γ* pathway [31]. In addition, positive ALK immunostaining targeting this activated fusion protein serves as a reliable marker for IMT, as performed in our case [3].

ALK protein is a RTK family member with an extracellular, transmembrane, and intracellular domain (ICD). The ICD of ALK mainly comprises a tyrosine kinase domain and the juxtamembrane region. The ALK kinase domain directly conducts enzyme catalysis, and this is where ALK inhibitors usually bind [31]. The production of ALK fusion proteins necessitates that the *ALK* gene breakpoint includes the entire tyrosine kinase domain [32], which is typically encoded by Exons 22–25 [33]. Interestingly, ALK fusions are identified in most lung cancers, and ALCLs occur at the beginning of Exon 20 of the *ALK* gene. However, Exon 19 encodes the transmembrane domain of oncoproteins. Exon 19-containing *ALK* fusions have been rare in lung cancers and other ALK-positive tumors. It has been reported more commonly in uterine IMTs, including pregnancy-associated IMTs. Eight among these were uterine IMTs with THBS1::ALK fusion (Table 2), as in our case [33–35]. This fusion retains the ALK kinase domain. The fusion of *THBS1* to *ALK* potentially results in increased expression and constitutive activation of the ALK kinase domain. For these cases, wherever fusion data was available, *THBS1* was fused at Exon 4, whereas our case showed fusion at Exon 7 [33]. One case of uterine IMT showed Exon 7 of *THBS1* fusion with Exon 18 of *ALK*, another rare sequence [35]. However, the significance of transmembrane domain preservation is still unknown and is a topic for further exploration. One case of uterine IMT harboring THSB1::ALK fusion showed a distinct strong cytoplasmic staining pattern with IHC [34]. A previously reported laryngeal IMT harboring this particular mutation also has similar strong cytoplasmic staining [29]. Further studies are required to investigate whether the distinctive ALK staining pattern is coincidental or associated with this specific fusion.

The other fusion partner, the *THBS1* gene, is located on 15q14 (Figure 3), is 16,393 bases in size, and is composed of 22 exons. Exons 2–21 encode the 5729b mRNA [36]. THBS1 encoded by *THBS1* is a matricellular adhesive glycoprotein that mediates cell-to-cell and cell-to-matrix interactions [37]. It also regulates intracellular signaling and extracellular matrix remodeling and is involved in physiological processes such as inflammation, angiogenesis, and tissue remodeling [37]. When secreted, it is involved in regulating platelet adhesion and angiogenesis [33]. *THBS1* has been previously identified as a 5′ fusion partner of *ALK* in IMT of the larynx (one case) [29] and uterus (five cases) [33, 35]. It is also reported to cause upregulation of ADGFR in acral fibrochondromyxoid tumor (AFCMT). Interestingly, the breakpoint Exon 21 in the AFCMT series led to protein conservation in AFCMT. Bouvier et al. have mentioned this *THBS1* expression as a source of excessive matrix in AFCMT [38]. As a key organizer of the ECM, *THBS1* involvement may contribute to the abundant matrix observed in the hypocellular myxoid pattern of IMT. In our case, the breakpoint for *THBS1* was at Exon 7, making functional protein production unlikely and hence minimal myxoid stroma.

The most common fusions identified in laryngeal IMTs are *TIMP3-ALK* (6/8) [39], *KIF5B-ALK* (1/8), and *THBS1-ALK* (1/8) prior to this study [7, 29, 39]. Genomic alterations affecting *ROS1* (10%), *PDGFRβ*, *RET*, and *NTRK1* have been detected in a subgroup of tumors that lack *ALK* rearrangements in other locations [29, 40, 41]. ROS1 and PDGFR*β* are actionable kinase fusions that hold the potential to improve management with targeted therapies [41]. An important point to note is that diagnosing ALK-negative IMT is challenging due to its markedly variable histomorphology, the absence of a definitive immunoprofile, and a long list of differential diagnoses related to the tumor's location [40]. Regardless of the type of fusion, no published study has reported molecular findings in a case of recurrent laryngeal IMT to date.

According to previous limited studies, in patients aged 40 years or older, *ALK* rearrangements are rare [7]. These *ALK*-negative cases are associated with an increased likelihood of mortality attributed to disease progression and distant metastasis [9]. Now, with advanced testing techniques, ALK positivity is increasingly seen in the older population [7, 33, 42]. The limited availability of ALK IHC in previously reported cases makes it difficult to draw definitive conclusions regarding the association between ALK1 status and tumor recurrence in laryngeal IMT [21]. In addition, while IHC is a useful screening tool, it does not offer any information regarding fusion partners [32]. The data is controversial regarding ALK-positive IMTs. The expression of ALK1 in pediatric IMT has been linked to recurrence [9, 14]. Conversely, some studies suggest an overall better prognosis in ALK1-positive cases [14, 27].

Nevertheless, surgical excision remains the primary treatment for laryngeal IMT, providing a favorable prognosis [18]. The challenging location of laryngeal IMTs makes complete removal difficult, resulting in a higher recurrence rate than other sites. Radiotherapy, steroids, and chemotherapy have been used previously in nonoperable cases and recurrences [43]. As most laryngeal IMTs harbor kinase fusions, targeting these fusions, particularly through ALK1 inhibition, represents a promising avenue for advancing personalized treatment and molecular-targeted therapy. Several studies report successful treatment of ALK-positive IMTs with crizotinib (a novel ALK tyrosine kinase inhibitor [TKI]) in other organs [20, 44–46] and the larynx [20, 46]. Also, Childress et al. and Xiang et al. have clearly demonstrated that the 5′ fusion partner affects the biochemical and cellular properties of the ALK fusion protein, including kinase activity, protein stability, and transformative potential, as well as the response to ALK TKIs [28, 32]. This approach has been successful in other ALK-positive tumors and could have significant clinical implications. Further study is necessary due to limited data caused by publication bias, complex treatment regimens, and variable follow-up time [18].

In short, we report the first case of recurrent laryngeal IMT in a 24-year-old patient with a novel THBS1::ALK1 fusion, emphasizing the significance of correlating molecular findings with tumor morphology and recurrent behavior. Our literature review on identifying kinase fusions in laryngeal IMTs highlights the importance of molecular profiling in enhancing diagnostic precision and providing diverse therapeutic avenues for patients [7].

## 5. Conclusion

In summary, we report a case of recurrent laryngeal IMT in a young patient carrying a novel THBS1::ALK fusion. Our findings expand the clinical and molecular spectrum of laryngeal IMTs and provide a better understanding of the *ALK* fusion partners found so far. It may open the door to the causation of recurrent IMTs and treatment options. The prompt and accurate reporting of novel fusion events and associated tumor characteristics is essential to facilitating improved treatment and prevention strategies.

## Figures and Tables

**Table 1:** Recurrent laryngeal inflammatory myofibroblastic tumors in literature.

**Study**	**Age/gender**	**Location**	**Treatment for primary tumor**	**Recurrence time frame**	**Treatment for recurrence**
Corsi et al. [23]	57/M	Anterior glottis commissure 0.8 cm, recurrence 0.6 cm	Fiberoptic laryngoscopic excision	2 months	Fiberoptic laryngoscopic excision
Wenig et al. [4]	65/M	Left true vocal cord and anterior commissure, 3 cm	Right cordectomy	1st recurrence—7 months 2nd recurrence—13 months	1st recurrence—laser excision (5000 cGy) plus radiotherapy 2nd recurrence—total laryngectomy
Alhumaid et al. [10]	54/M	Left true vocal fold and subglottic mass, 1.5 cm	Microlaryngoscopy with excisional biopsies	1 year	Endoscopic resection with laser excision
Völker et al. [24]	34/F	Right vocal cord, 0.8 cm; recurrence 1.2 cm	Microlaryngoscopy with complete excision	3 months	Complete resection
Da et al. [12]	9/M	Glottis	Extensive excision	4 months	Extended resection under electronic laryngoscope
He, Dong, and Liu [14]	10/M	Right vocal cord, anterior commissure, and anterior third of left vocal cord	Direct microlaryngoscopy with CO_2_ laser	1st recurrence—10 months 2nd recurrence—6 months 3rd recurrence—5 months 4th recurrence—2 months	Multiple resections, the last one with negative margins on frozen sections
Kumar et al. [15]	6/M	Right vocal cord	Endolaryngeal excision	1st recurrence—4 months 2nd recurrence—6 months	1st recurrence—endolaryngeal reexcision with tracheostomy 2nd recurrence—laser excision and oral celecoxib
Do et al. [25]	37/M	Right vocal cord	Suspension microlaryngoscopy and cold steel biopsy	A few months	Oral steroids
Rodrigues et al. [26]	2/M	Aryepiglottic fold	Endoscopic excision	N/A	Endoscopic partial supra-glottic laryngectomy
Mella, Hakim, and Fakhoury [27]	56/M	Right vocal cord—5 mm	Laryngoscopy and cold steel excision + radiotherapy	Multiple recurrences	Multiple excisions, with total laryngectomy for the last recurrence
Our case	24/M	Right vocal cord	Microlaryngoscopic excisional biopsy with Kenalog injection	13 months	Microlaryngoscopic excisional biopsy with Kenalog injection

*Note:* The cases where incomplete resection was clearly mentioned were excluded as recurrent in the table [13].

Abbreviations: F: female, M: male, N/A: not available.

**Table 2:** THBS1::ALK fusions in the literature.

**Study**	**Age/gender**	**Organ**	**Exonic breakpoint 3 **′**gene partner**	**Exonic breakpoint 5 **′**gene partner**	**Prognosis**	**Follow-up (months)**
Our case	24/M	Larynx	19	7	LR	16 months
Elktaibi et al. [29]	23/M	Larynx	NA	NA	NED	6 months
Kerr et al. [7]	10/M	Oropharynx	19	7	NED	42 months
Haimes et al. [33]	47/F	Uterus	19	4	NED (hysterectomy)	93 months
Haimes et al. [33]	46/F	Uterus	19	4	NED (myomectomy)	36 months
Haimes et al. [33]	28/F	Uterus	19	4	NED (myomectomy)	35 months
Devereaux et al. [35]	23/F	Pregnant uterus	19	4	Expelled postpartum	26 months
Devereaux et al. [35]	20/F	Pregnant uterus	18	7	Expelled at VD and hysterectomy	8 months
Bennett et al. [34]	28/F	Uterus	NA	NA	NED	7 months
Bennett et al. [34]	38/F	Uterus	NA	NA	NED	5 months
Bennett et al. [34]	28/F	Uterus	NA	NA	NA	NA

Abbreviations: LR: local recurrence, NA: not available, NED: no evidence of disease, VD: vaginal delivery.

## Data Availability

Data is available for review on request.

## References

[1] Colizza A., Meliante P. G., Donsante S. (2022). Inflammatory myofibroblastic tumour of the larynx: report of a case. *Ear, Nose, & Throat Journal*.

[2] Hornick J. L., Yamamoto H. (2020). Inflammatory myofibroblastic tumour. *WHO Classification of Tumours Editorial Board. Soft tissue and bone tumours*.

[3] Casanova M., Brennan B., Alaggio R. (2020). Inflammatory myofibroblastic tumor: the experience of the European pediatric Soft Tissue Sarcoma Study Group (EpSSG). *European Journal of Cancer*.

[4] Wenig B. M., Devaney K., Bisceglia M. (1995). Inflammatory myofibroblastic tumor of the larynx. A clinicopathologic study of eight cases simulating a malignant spindle cell neoplasm. *Cancer*.

[5] Cantera J. E., Alfaro M. P., Rafart D. C. (2015). Inflammatory myofibroblastic tumours: a pictorial review. *Insights into Imaging*.

[6] Palaskar S., Koshti S., Maralingannavar M., Bartake A. (2011). Inflammatory myofibroblastic tumor. *Contemporary Clinical Dentistry*.

[7] Kerr D. A., Thompson L. D. R., Tafe L. J. (2021). Clinicopathologic and genomic characterization of inflammatory myofibroblastic tumors of the head and neck: highlighting a novel fusion and potential diagnostic pitfall. *The American Journal of Surgical Pathology*.

[8] Chen Y. F., Zhang W. D., Wu M. W., Ou-Yang D., Zhang Q. (2011). Inflammatory myofibroblastic tumor of the head and neck. *Medical Oncology*.

[9] Coffin C. M., Watterson J., Priest J. R., Dehner L. P. (1995). Extrapulmonary inflammatory myofibroblastic tumor (inflammatory pseudotumor). A clinicopathologic and immunohistochemical study of 84 cases. *The American Journal of Surgical Pathology*.

[10] Alhumaid H., Bukhari M., Rikabi A. (2011). Laryngeal myofibroblastic tumor: case series and literature review. *International Journal of Health Sciences*.

[11] Tay S. Y., Balakrishnan A. (2016). Laryngeal inflammatory myofibroblastic tumor (IMT): a case report and review of the literature. *Journal of Medical Case Reports*.

[12] Da M., Qian B., Mo X. (2021). Inflammatory myofibroblastic tumors in children: a clinical retrospective study on 19 cases. *Frontiers in Pediatrics*.

[13] Hanna S. J., Blenke E., Sharma R., Knight L. C. (2005). Laryngeal inflammatory pseudotumour: an unusual cause of airway obstruction. *International Journal of Pediatric Otorhinolaryngology*.

[14] He C. Y., Dong G. H., Liu H. G. (2014). Recurrent laryngeal inflammatory myofibroblastic tumor with positive anaplastic lymphoma kinase mimicking recurrent respiratory papillomatosis: a case report. *World Journal of Surgical Oncology*.

[15] Kumar N., Saravanamuthu T., Srinivasan A., Raman T., Scott J. X. (2018). Pediatric inflammatory myofibroblastic tumors of the airway: two case reports with varying clinical presentation. *Iranian Journal of Otorhinolaryngology*.

[16] Kaytez S. K., Kavuzlu A., Oguz H. (2021). Laryngeal inflammatory myofibroblastic tumor with anemia and thrombocytosis. *Ear, Nose, & Throat Journal*.

[17] Guilemany J. M., Alós L., Alobid I., Bernal-Sprekelsen M., Cardesa A. (2005). Inflammatory myofibroblastic tumor in the larynx: clinicopathologic features and histogenesis. *Acta Oto-Laryngologica*.

[18] Foster H. J., Ow T. J., Bottalico D., Scott G., Ustun B., Shifteh K. (2021). Inflammatory myofibroblastic tumor of the larynx: case report. *Clinical Case Reports*.

[19] Devaney K. O., Lafeir D. J., Triantafyllou A. (2012). Inflammatory myofibroblastic tumors of the head and neck: evaluation of clinicopathologic and prognostic features. *European Archives of Oto-Rhino-Laryngology*.

[20] Lahlou G., Classe M., Wassef M. (2017). Sinonasal inflammatory myofibroblastic tumor with *anaplastic lymphoma kinase 1* rearrangement: case study and literature review. *Head and Neck Pathology*.

[21] Smaily H., Cherfane P., Matar N. (2021). Pediatric laryngeal inflammatory myofibroblastic tumour: case report and systematic review of the literature. *Auris, Nasus, Larynx*.

[22] Dava C. J., Hajiioannou J. K., Terzis A., Bizakis J. (2012). An inflammatory pseudotumour of the larynx: a case report and literature review of an unusual tumour. *Ecancermedicalscience*.

[23] Corsi A., Ciofalo A., Leonardi M., Zambetti G., Bosman C. (1997). Recurrent inflammatory myofibroblastic tumor of the glottis mimicking malignancy. *American Journal of Otolaryngology*.

[24] Völker H. U., Scheich M., Höller S. (2007). Differential diagnosis of laryngeal spindle cell carcinoma and inflammatory myofibroblastic tumor–report of two cases with similar morphology. *Diagnostic Pathology*.

[25] Do B. A., Varshney R., Zawawi F., Levental M., Caglar D., Young J. (2014). Inflammatory myofibroblastic tumor of the larynx-a case report. *Journal of Voice*.

[26] Rodrigues M., Taylor R. J., Sun C. C., Wolf J. S. (2005). Inflammatory myofibroblastic tumor of the larynx in a 2-year-old male. *ORL: Journal for Otorhinolaryngology and Its Related Specialties*.

[27] Mella M. D., Hakim E., Fakhoury R. (2021). Aggressive benign laryngeal mass: inflammatory fibroblastic tumor. *Otolaryngology Case Reports*.

[28] Childress M. A., Himmelberg S. M., Chen H., Deng W., Davies M. A., Lovly C. M. (2018). ALK fusion partners impact response to ALK inhibition: differential effects on sensitivity, cellular phenotypes, and biochemical properties. *Molecular Cancer Research*.

[29] Elktaibi A., Benzerdjeb N., Ameur F., Daveau C., Tantot J., Costes M. V. (2020). A novel *ALK-THBS1* fusion in a laryngeal inflammatory myofibroblastic tumour: a case report and literature review. *Head and Neck Pathology*.

[30] Antonescu C. R., Suurmeijer A. J., Zhang L. (2015). Molecular characterization of inflammatory myofibroblastic tumors with frequent ALK and ROS1 gene fusions and rare novel RET rearrangement. *The American Journal of Surgical Pathology*.

[31] Huang H. (2018). Anaplastic lymphoma kinase (ALK) receptor tyrosine kinase: a catalytic receptor with many faces. *International Journal of Molecular Sciences*.

[32] Xiang Y., Zhang S., Fang X. (2022). Therapeutic advances of rare ALK fusions in non-small cell lung cancer. *Current Oncology*.

[33] Haimes J. D., Stewart C. J. R., Kudlow B. A. (2017). Uterine inflammatory myofibroblastic tumors frequently harbor ALK fusions with IGFBP5 and THBS1. *The American Journal of Surgical Pathology*.

[34] Bennett J. A., Nardi V., Rouzbahman M., Morales-Oyarvide V., Nielsen G. P., Oliva E. (2017). Inflammatory myofibroblastic tumor of the uterus: a clinicopathological, immunohistochemical, and molecular analysis of 13 cases highlighting their broad morphologic spectrum. *Modern Pathology*.

[35] Devereaux K. A., Fitzpatrick M. B., Hartinger S., Jones C., Kunder C. A., Longacre T. A. (2020). Pregnancy-associated inflammatory myofibroblastic tumors of the uterus are clinically distinct and highly enriched for TIMP3-ALK and THBS1-ALK fusions. *The American Journal of Surgical Pathology*.

[36] Isenberg J. S., Roberts D. D. (2020). THBS1 (thrombospondin-1). *Atlas of Genetics and Cytogenetics in Oncology and Haematology*.

[37] Duan F. M., Fu L. J., Wang Y. H. (2021). THBS1 regulates trophoblast fusion through a CD36-dependent inhibition of cAMP, and its upregulation participates in preeclampsia. *Genes & Diseases*.

[38] Bouvier C., Le Loarer F., Macagno N. (2020). Recurrent novel THBS1-ADGRF5 gene fusion in a new tumor subtype "acral fibrochondromyxoid tumors". *Modern Pathology*.

[39] Yorita K., Togashi Y., Nakagawa H. (2019). Vocal cord inflammatory myofibroblastic tumor with mucoid deposits harboring TIMP3-ALK fusion: a potential diagnostic pitfall. *Pathology International*.

[40] Debonis S. A., Bongiovanni A., Pieri F. (2021). ALK-negative lung inflammatory myofibroblastic tumor in a young adult: a case report and literature review of molecular alterations. *Medicine*.

[41] Lovly C. M., Gupta A., Lipson D. (2014). Inflammatory myofibroblastic tumors harbor multiple potentially actionable kinase fusions. *Cancer Discovery*.

[42] Pierry C., Pérot G., Karanian-Philippe M. (2015). Polypoid laryngeal inflammatory myofibroblastic tumors: misleading lesions: description of six cases showing ALK overexpression. *American Journal of Clinical Pathology*.

[43] Zitsch 3rd R. P., Pollak N., Loy T. S. (2007). Management of inflammatory pseudotumor of the larynx. *Otolaryngology—Head and Neck Surgery*.

[44] Jacob S. V., Reith J. D., Kojima A. Y., Williams W. D., Liu C., Vila Duckworth L. (2014). An Unusual Case of Systemic Inflammatory Myofibroblastic Tumor with Successful Treatment with ALK‐Inhibitor. *Case Reports in Pathology*.

[45] Butrynski J. E., D'Adamo D. R., Hornick J. L. (2010). Crizotinib in *ALK*-rearranged inflammatory myofibroblastic tumor. *New England Journal of Medicine*.

[46] Theilen T. M., Soerensen J., Bochennek K. (2018). Crizotinib in ALK^+^ inflammatory myofibroblastic tumors–current experience and future perspectives. *Pediatric Blood & Cancer*.

